# Willingness and skills among students from non-health academic fields in providing efficient basic life support

**DOI:** 10.1016/j.clinsp.2024.100518

**Published:** 2024-11-08

**Authors:** Perola Nakandakari Sugimoto, Gabriela Buno Gouvêa, Igor Caitano Salles, Heráclito Barbosa de Carvalho, Priscila Aikawa, Liana Maria Torres de Araújo Azi, Luiz Fernando Ferraz da Silva, Mariangela Macchione, Federico Semeraro, Andrew Lockey, Robert Greif, Maria José Carvalho Carmona, Bernd Walter Böttiger, Naomi Kondo Nakagawa

**Affiliations:** a Education, Assessment and Intervention in Cardiovascular Group, Faculdade de Medicina da Universidade de São Paulo (FMUSP), São Paulo, SP, Brazil; b Preventive Department, Faculdade de Medicina da Universidade de São Paulo (FMUSP), São Paulo, SP, Brazil; c Institute of Biological Sciences, Universidade Federal do Rio Grande do Sul (UFRGS), Rio Grande do Sul, RS, Brazil; d Federal University of Bahia, Bahia, BA, Brazil; e Department of Anesthesia, Intensive Care and Prehospital Emergency, Maggiore Hospital Carlo Alberto Pizzardi, Bologna, Italy; f Calderdale and Huddersfield NHS Trust, Halifax, United Kingdom; g School of Medicine, Sigmund Freud University Vienna, Vienna, Austria; h University of Bern, Bern, Switzerland; i Anesthesiology Discipline, Faculdade de Medicina da Universidade de São Paulo (FMUSP), São Paulo, SP, Brazil; j University of Cologne, Department of Anaesthesiology and Intensive Care Medicine, University Hospital, Medical Faculty, Germany; k Department of Pathology, Faculdade de Medicina da Universidade de São Paulo (FMUSP), São Paulo, SP, Brazil

**Keywords:** Sudden cardiac arrest, Cardiopulmonary resuscitation, Basic life support, Acute myocardial infarction, Medical students, Automated external defibrillator

## Abstract

Education in basic life support is widely proposed to increase survival in out-of-hospital sudden cardiac arrest. The authors aimed to assess knowledge, skills, and attitudes, including willingness to help, regarding myocardial infarction and sudden cardiac arrest among university students of all fields of knowledge.

**Methods:**

An Ethical Research Committee approved this cross-sectional study. An electronic survey “KIDS SAVE LIVES BRAZIL” was sent to 58,862 students of 82 disciplines in three universities, aged ≥ 18 years. The survey covered three categories: knowledge, skills, and attitude. Each category was graded between 0 and 10 points (the highest).

**Results:**

Among students, 4,803 undergraduates (8.2 %) answered the survey, and were divided into three groups of disciplines: medicine (219, ∼21.7 years, 38 % male), other-healthcare (*n* = 1,058; ∼22.9 years; 36 % male), and non-health-care (*n* = 3,526; ∼22.9 years; 35 % male). All three groups showed significant differences between them (*p* < 0.001). The non-health-care compared with medicine and other healthcare groups showed, respectively, the lowest median scores (25 %‒75 %) in knowledge (4.0 [0.0‒9.3], 4.0 [4.0‒8.0], and 4.0 [4.0‒4.7]), skills (2.4 [1.2‒3.3], 6.4 [4.0‒8.3], 4.0 [2.4‒6.2]), and attitude (5.9 [5.9‒6.8], 7.3 [5.9‒7.3], and 7.3 [5.9‒7.3]).

**Conclusion:**

University students who answered the e-survey have the willingness to help victims suffering from myocardial infarction or sustaining sudden cardiac arrest. However, non healthcare students markedly lack the knowledge and skills to perform cardiopulmonary resuscitation and automated external defibrillation. These findings reveal a stark difference in basic life support competencies between students in related healthcare fields and those in non-health fields, emphasizing the need for universal basic life support training.

## Highlights

• These findings reveal a stark difference in basic life support competencies between students in health-care related fields and those in non-health fields, emphasizing the need for universal basic life support training.
• An action for curriculum modification to include basic life support training for all students is timely and practical, given the global burden of heart disease and the proven benefits of early intervention in sudden cardiac arrest cases.
• This study contributes significantly to the ongoing discussion about health education and the role of non-health professionals in emergency medical response. It may serve as a catalyst for policy changes within educational institutions and among healthcare policymakers, aiming to create a more resilient and responsive community in the face of out-of-hospital medical emergencies.

## Introduction

Acute Myocardial Infarction (AMI) can be a cause of arrhythmia and Out-of-Hospital Sudden Cardiac Arrest (OHCA) in adults.^1^ Both, AMI and OHCA, are major public health burdens with high incidence, high morbidity and high mortality rates worldwide.^2^^,^^3^ Survival rates after OHCA are globally low (< 10 %), and are proportional to the rate of people trained in early high-quality Cardiopulmonary Resuscitation (CPR), and adequate use of an Automated External Defibrillator (AED).^3^, ^4^, ^5^

In the last two decades, studies showed that geographical regions, racial distributions, bystanders aged > 30 years, and low income are associated with lower probabilities of bystanders initiating CPR in OHCA.^3^^,^^6^^,^^7^ Recently, education for laypeople in CPR and AED use has been widely proposed since childhood to improve quality of life^8^ and to increase survival after OHCA by the World Health Organization,^9^ and the International Liaison Committee on Resuscitation.^10^^,^^11^

BLS training follows the current international guidelines and young adults have favorable cognitive and physical characteristics (larger than 1.5 m, >50 kg, or body mass index higher than 22 kg.*m*^−2^ to adequately perform CPR and to use AED.^12^^,^^13^ Undergraduate students of medicine,^14^, ^15^, ^16^ nursing, physiotherapy^17^ have BLS included in their curriculum. However, little is known about non-healthcare university students’ competencies in BLS, and their willingness to help people victims of AMI and OHCA. Whether the authors find a positive attitude to help people, it will be important to know if they have knowledge, skills and attitudes to identify and take first actions in these situations.

This study is based on an electronic survey and aimed to investigate self-rated willingness to help victims, knowledge, skills and attitudes on AMI and OHCA among university students of all disciplines divided into medicine, other health-care disciplines, and nonhealth-care disciplines.

## Methods

This is a cross-sectional observational study of the KIDS SAVE LIVES BRAZIL project with approval of the Ethical Committee of the Faculty of Medicine, São Paulo University, Brazil (CAAE: 25,218,819.0.0000.0065). The authors recruited students from 82 different higher education disciplines, aged ≥ 18 years, both sexes, from three public universities in Brazil (University of São Paulo, State University of Campinas, and Federal University of Rio Grande). An electronic survey was sent only once to all students by the Rectory of the University, from March 2020 to February 2022. The authors included students who agreed with the research written informed consent, and who answered the survey until April 2022. The authors excluded those students who did not provide information on age or university discipline. Students were allocated to the following three groups of disciplines: medicine, other healthcare, and non-healthcare.

The authors performed a sample size calculation by estimating a population proportion of 15 % that would grade at least five in knowledge with a specified absolute proportion of 1 %, which resulted in 4898 students. The formula was^18^

z21−α/2(1−p)

n1=−−−−−−−−−−−−−−.

E2p

Where: n_1_ is the estimated sample size; E is the assumed level of confidence of 95 %; *z* is the z curve area for different levels of significance; and p is the expected proportion for the event.

### Electronic questionnaire

Several aspects of the present survey were based on previous work^19^ which aimed to understand the scenario in primary, secondary and high schools on key principles of KIDS SAVE LIVES BRAZIL. The authors created an electronic survey in the Google form platform, that was available at the University. Each student performed a self-rating answer. It is a 25-item multiple choice online questionnaire covering three categories: knowledge (5-items), skills (8-items), and attitude (12-items) towards OHCA and AMI. Each category (knowledge, skills and attitude) was graded between zero and 10 (the highest grade). The questions included in each category are in the Supplementary Material.

### Statistical analysis

The authors used the Statistical Software STATA for Windows (Stata Corp LLC. Texas, USA), version 24.

The authors applied the Shapiro-Wilk Test for normal distribution analysis of age and grades in each category (knowledge, skills and attitude).

Data from the three groups of disciplines: medicine, other-healthcare, and non healthcare are presented as means (± Standard Deviation) if continuous data show normal distributions, and they were analyzed using the ANOVA Test. Data with non-normal distributions are presented as medians (25 %‒75 % quartiles), and were analyzed using the Kruskal-Wallis Test. When appropriate, a multiple comparison paired analysis was performed using the Dunn's Test.

Categorical variables are presented as numbers and proportions. The categorical variables were sex and grades above (yes/no) and the median score of all students of all disciplines in each category (knowledge, skills, and attitude). The authors used the Chi-Squared Test to analyze data among the three groups.

A p-value < 0.05 was considered statistically significant.

## Results

**Fig. 1:**
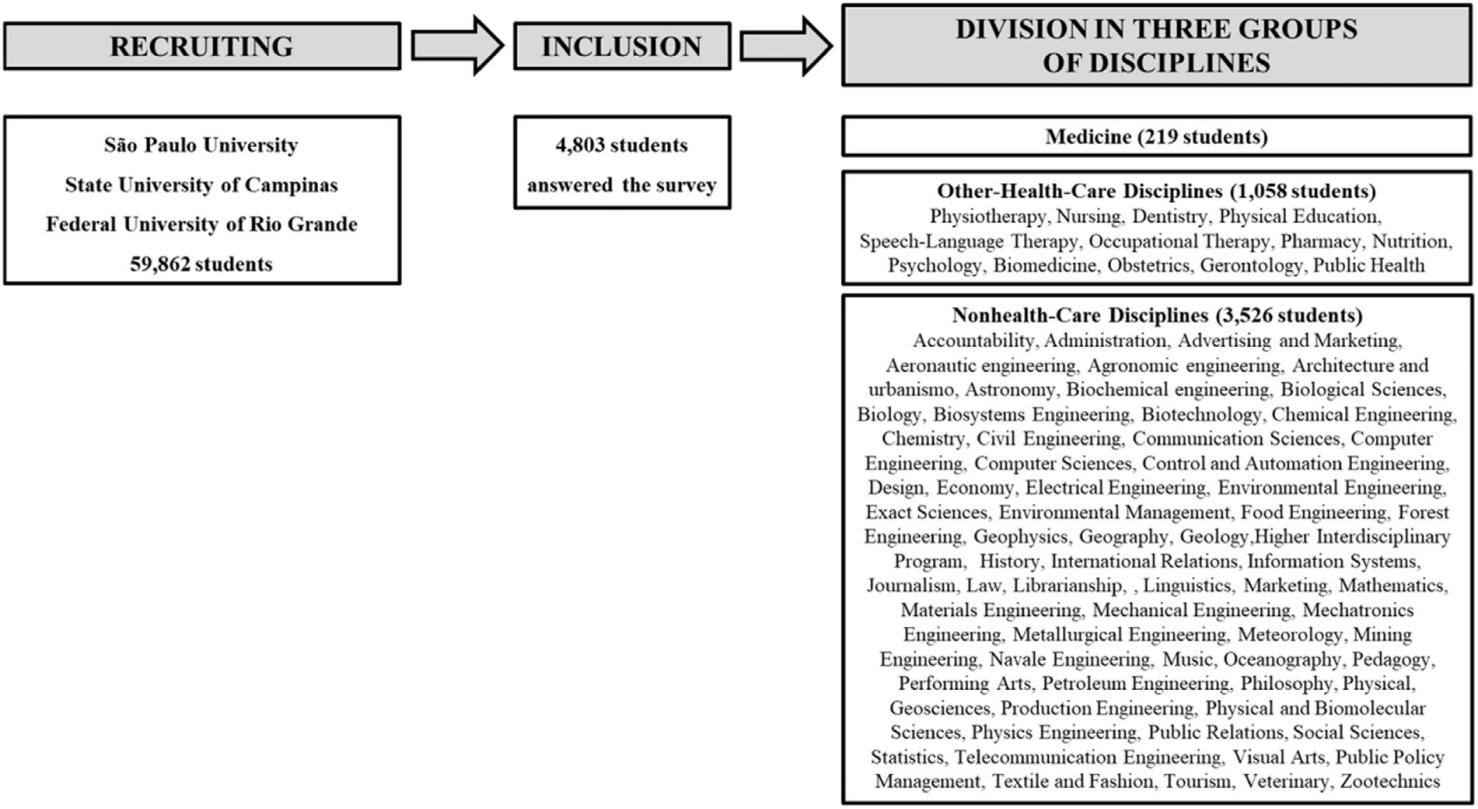
The cross-over study design using an electronic survey sent to all students of all years of three Universities. Responders from 82 disciplines were divided into three groups of disciplines: medicine, other-healthcare, and non healthcare.

The authors sent the electronic survey to 58,862 undergraduates of 82 disciplines from three public Universities (Fig. 1). A total of 4803 students (8.2 %) answered the survey: 5 % of the students were studying medicine (*n* = 219), 22 % was studying other-health-care disciplines (*n* = 1058), and 73 % was studying non healthcare disciplines (*n* = 3526). There were 1.645 students who had a BLS course before this survey. Among them, 145 were medical students, 242 were other-health-care students, and 1258 were nonhealthy-care students. The other 3158 university students did not have it (74 medical students, 816 healthcare students, and 2268 non healthcare students).

Among 4803 respondents, 98 % (*n* = 4708) showed a willingness to help people in SCA. Similarly, 98 % of respondents (*n* = 4747) wished to have a practical BLS course at school.

Students in medicine were younger than students of the other healthcare and non healthcare disciplines (Table 1). Female students were prevalent in this study. However, sex proportions were similar between the three groups of disciplines.

**Table 1:** Demographic characteristics are presented as mean values and standard deviations or numbers of subjects and proportions. Comparisons between groups of disciplines (medicine, other-healthcare, and nonhealth-care) were performed using the ANOVA test or the Chi-Squared Test, when appropriate

	Medicine (*n* = 219)	Other-health-care (*n* = 1058)	Nonhealth-care (*n* = 3526)	p-value
**Age, years**	21.7 ± 3.3	22.9 ± 6.7	22.9 ± 6.5	0.045
**Sex, n (%)**				0.408
Male	83 (37.9)	383 (36.2)	1218 (34.5)	
Female	136 (62.1)	675 (63.8)	2308 (65.5)	

**Fig. 2:**
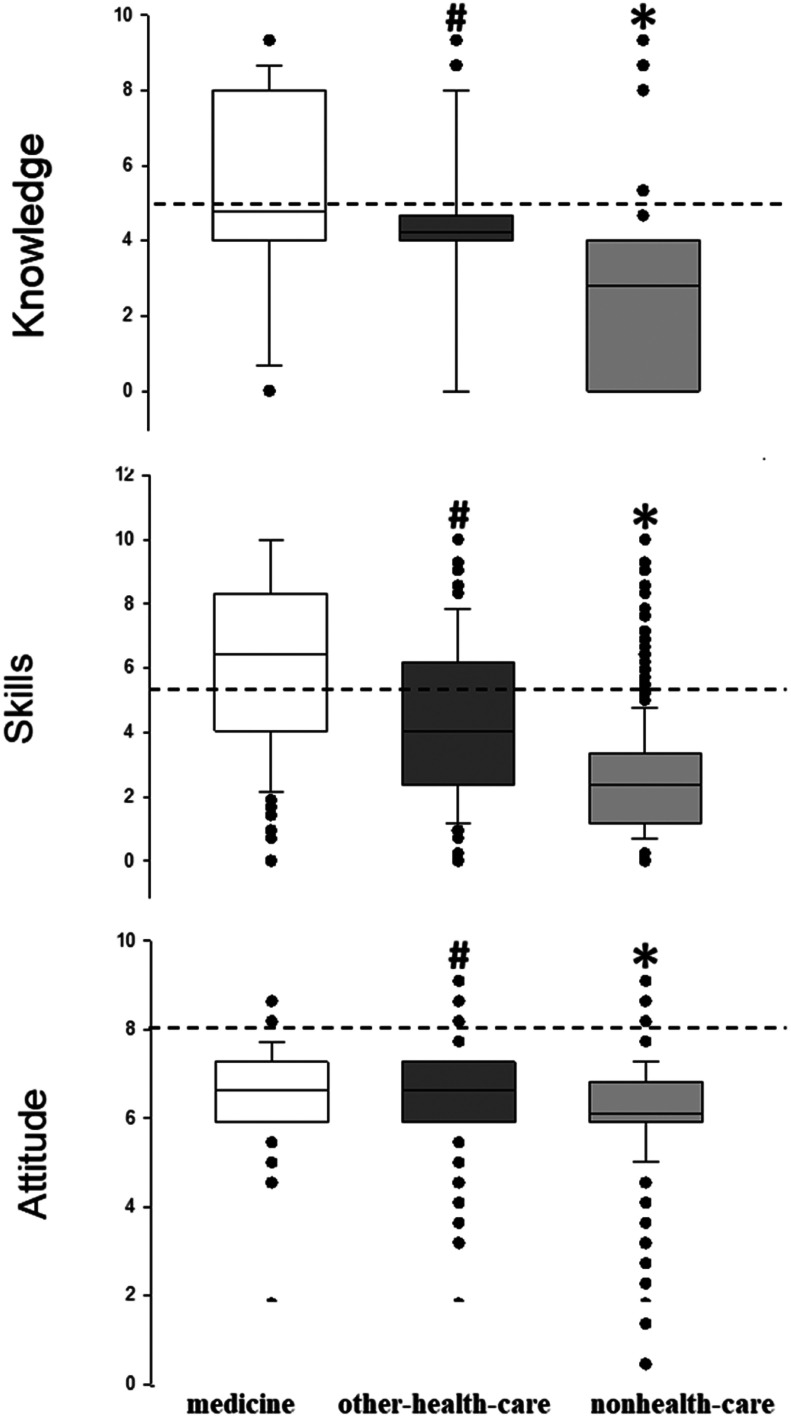
Boxplot of self-reported grades in three categories (knowledge, skills and attitude) by university students of medicine (□), other-health-care disciplines (▀) and nonhealth-care disciplines (▀). Dashed lines show the median values of all students in each category. Significant differences were found between groups using the Kruskall-Wallis Test with post-hoc analysis (Dunn's Test) (* *p* < 0.001 vs. other groups; # *p* < 0.001 vs. medicine).

Grades in the category's knowledge, skills and attitude are presented in Fig. 2. The median score (25 %‒75 % quartiles) of all students of all disciplines in knowledge was 3.2 points (0‒4.0). After post-hoc analysis with the Dunn's Test, the non healthcare students showed the lowest (*p* < 0.001) median score (25 %‒75 % quartiles) of 4.0 (0‒9.3) than medical students with a median score of 4.0 (4.0‒8.0) and the other-health-care students with a median score of 4.0 points (4.0‒4.7).

The median score (25 %‒75 % quartiles) of perceived skills reported by all students of all disciplines was 3.1 (1.7‒4.1). The three disciplines showed significantly different median scores between them in skills (*p* < 0.001). Medical students had the highest score of 6.4 (4.0‒8.3). The other healthcare students had 4.0 (2.4‒6.2). The non healthcare group obtained the lowest score of 2.4 (1.2‒3.3) (Fig. 2).

The median score (25 %‒75 % quartiles) in attitude reported by all students of all disciplines was 6.2 (5.9‒7.3) and there were significant differences between all three groups (*p* < 0.001). The non healthcare university students had 6.1 points (5.9‒6.8) lower than the medicine group which had 6.6 points (5.9‒7.3), and the other-health-care disciplines with 6.6 points (5.9‒7.3) (Fig. 2).

In the analysis of the proportions of students that graded above the median score in each category (Table 2), the authors found that non healthcare disciplines had reduced proportions of students compared with the medical and other healthcare disciplines in two categories knowledge and skills.

**Table 2:** Numbers and proportions of students with grades above the median score of all students of all disciplines in each category (knowledge, skills and attitude).

	Medicine (*n* = 219)	Other-health-care (*n* = 1058)	Nonhealth-care (*n* = 3526)	p-value
**Knowledge**	65 (29.7)	295 (27.9)	160 (4.5)^a^	<0.001
**Skills**	185 (84.5)	687 (64.9)^b^	1298 (36.8)^a^	<0.001
**Attitude**	150 (68.5)	594 (56.1)	1088 (30.9)^a^	<0.001

Comparisons between groups of disciplines (medicine, other-healthcare, and nonhealth-care) were performed using the Chi-Squared Test (^a^, vs. the other groups,^b^, vs. medicine).

## Discussion

To our knowledge, this is the first study investigating self-rated knowledge, skills and attitudes in SCA and AMI among students of 82 different disciplines in multiple higher education institutions of a lower resource country. Students (aged about 23 years and 65 % female) were grouped into three groups of disciplines: Medicine, Other-healthcare and Nonhealth-care. The present study reveals that independently of healthcare or non healthcare fields, the authors found significant differences in BLS knowledge, skills and attitude between students of healthcare related disciplines and non healthcare disciplines. Additionally, the great majority (98 %) of university students of all disciplines reported a willingness to help victims of SCA. Taken together, this study may emphasize the need for universal BLS training.

Interesting findings are in the attitude category. The authors had the same proportion of those who had the BLS course as those who did not have it (1:2), previously to this survey in the three groups. An important aspect found in the present study was that, independently of medicine, healthcare-related or non healthcare disciplines, the great majority of students (98 %) showed a willingness to help people in medical emergencies and wished to have a BLS course at school or university. However, students of non healthcare disciplines have nothing related to BLS theory or practice on why, what, and how to respond to save a life in their academic curricula at the university.^20^ University students are, in general, young adults with favorable cognitive and physical characteristics to perform BLS.^8^ Then, it is of note that the non healthcare students compose an important larger target group of society to develop competencies as first responders in out-of-hospital emergencies. Taken together, these results raise the need for curriculum modification by adding BLS training to students of all academic disciplines at higher education. University students should know why, what, and how to respond to out-of-hospital emergencies, taking first actions in SCA and AMI until the emergency medical service arrival. Each individual may have his/her own way of learning, and a great variety of educational modalities can be used for this purpose.^7^^,^^8^

The authors found significant differences between groups in knowledge, skills, and attitude. As expected, in the present study, medical students rated the highest scores in knowledge, skills and attitude facing SCA and AMI. The year of medical school and type of BLS training vary among countries and geographical regions,^20^ but most studies in the literature have been performed with medical students in their final year of school.^21^ Students in the 6th year of medical school may achieve higher grades as they are supposed to have had several opportunities along the six years curriculum for competencies (knowledge, skills and attitude) development to manage and deal with SCA and AMI. However, by including students of all six years of medical school, the authors intended to overcome this volunteer bias.

Students of other-health-care academic disciplines (all years of university) were also investigated. They rated lower median grades in knowledge, skills and attitude in SCA and AMI than medical students. This result can be explained by the fact that only a few disciplines (dentistry, nursing, and physiotherapy) also have BLS training, and other content on AMI, and SCA in their curriculum.^17^ Other healthcare disciplines included in this study, such as physical education, speech therapy and occupational therapy did not have BLS practical training in their curriculum.

The present study has limitations. The electronic survey on emergency situations was sent to approximately 59,000 students of three public universities. With only 8.2 % of respondents, one may argue that these results may not be extrapolated to the broader population. Indeed, the number of respondents (4803) was lower than that estimated (*n* = 4898). However, the authors studied several segments among students by inviting all students of the three public universities to participate in the survey. There is a national policy that reserves 50 % of the total entry places in public universities for students that passed the exams with low income, and/or blacks, and/or indians. In this context, the authors speculate that this sample of students in higher education would have a relative wide demographic and different backgrounds (low, middle and high socioeconomic status, different races, different school years, etc.) across multiple institutions. Another aspect is that this survey was not validated. The authors don´t know if it measures what is supposed to measure, and more studies are needed to assess its validity to generate similar results if repeated. Another limitation was that the authors used a self-rated and directive electronic survey, focusing on BLS skills and attitude, with no practical observations or objective measures. This is a study based on a self-rating online questionnaire that may induce bias as respondents tend to over-report normative behavior, and a directive survey may increase the likelihood of respondents to report certain behaviors that would appear socially agreeable.^22^ However, in this context, some aspects may help to overcome this bias, such as, the respondent identity was covered, the survey was not sent by a specific discipline, or related to a specific academic aspect.

Taken together, the present findings could contribute to the ongoing discussion about public health education and the role of non healthcare students and professionals in emergency medical response. This study calls for curriculum modification to include BLS training for all students of all disciplines in higher education. Nowadays, this can be considered timely and practical, given the global burden of heart disease and the proven benefits of early intervention in SCA cases. It may serve as part of a worldwide catalyst for policy changes within educational institutions and among healthcare policymakers, aiming to create a more resilient and responsive community in the face of out-of-hospital medical emergencies.

The present study reveals significant differences in BLS self-reported knowledge, skills, and attitude between students of health-care related fields and non healthcare disciplines, emphasizing the need for universal BLS training.

## Conclusion

The non healthcare university students have the willingness to help victims of AMI and OHCA, as students of medicine and other healthcare disciplines. However, the non healthcare students need to have the knowledge, develop skills, and improve attitudes to identify and take first actions in these situations.

## CRediT authorship contribution statement

**Perola Nakandakari Sugimoto:** Conceptualization, Formal analysis, Investigation, Data curation, Writing – original draft, Visualization. **Gabriela Buno Gouvêa:** Conceptualization, Formal analysis, Investigation, Data curation, Writing – original draft, Visualization. **Igor Caitano Salles:** Conceptualization, Formal analysis, Investigation, Data curation, Writing – original draft, Visualization. **Heráclito Barbosa de Carvalho:** Conceptualization, Methodology, Software, Validation, Formal analysis, Resources, Data curation, Writing – original draft, Writing – review & editing, Visualization, Supervision. **Priscila Aikawa:** Methodology, Writing – original draft, Writing – review & editing, Visualization, Supervision. **Liana Maria Torres de Araújo Azi:** Writing – review & editing. **Luiz Fernando Ferraz da Silva:** Methodology, Writing – review & editing. **Mariangela Macchione:** Methodology, Writing – original draft, Writing – review & editing, Visualization. **Federico Semeraro:** Writing – review & editing. **Andrew Lockey:** Writing – review & editing. **Robert Greif:** Writing – review & editing. **Maria José Carvalho Carmona:** Conceptualization, Methodology, Writing – original draft, Writing – review & editing, Visualization, Supervision, Project administration. **Bernd Walter Böttiger:** Writing – review & editing. **Naomi Kondo Nakagawa:** Conceptualization, Methodology, Validation, Formal analysis, Investigation, Resources, Data curation, Writing – original draft, Writing – review & editing, Visualization, Supervision, Project administration, Funding acquisition.

## CRediT authorship contribution statement

**Perola Nakandakari Sugimoto:** Conceptualization, Formal analysis, Investigation, Data curation, Writing – original draft, Visualization. **Gabriela Buno Gouvêa:** Conceptualization, Formal analysis, Investigation, Data curation, Writing – original draft, Visualization. **Igor Caitano Salles:** Conceptualization, Formal analysis, Investigation, Data curation, Writing – original draft, Visualization. **Heráclito Barbosa de Carvalho:** Conceptualization, Methodology, Software, Validation, Formal analysis, Resources, Data curation, Writing – original draft, Writing – review & editing, Visualization, Supervision. **Priscila Aikawa:** Methodology, Writing – original draft, Writing – review & editing, Visualization, Supervision. **Liana Maria Torres de Araújo Azi:** Writing – review & editing. **Luiz Fernando Ferraz da Silva:** Methodology, Writing – review & editing. **Mariangela Macchione:** Methodology, Writing – original draft, Writing – review & editing, Visualization. **Federico Semeraro:** Writing – review & editing. **Andrew Lockey:** Writing – review & editing. **Robert Greif:** Writing – review & editing. **Maria José Carvalho Carmona:** Conceptualization, Methodology, Writing – original draft, Writing – review & editing, Visualization, Supervision, Project administration. **Bernd Walter Böttiger:** Writing – review & editing. **Naomi Kondo Nakagawa:** Conceptualization, Methodology, Validation, Formal analysis, Investigation, Resources, Data curation, Writing – original draft, Writing – review & editing, Visualization, Supervision, Project administration, Funding acquisition.

## Conflicts of interest

Federico Semeraro is Chair-Elect of the European Resuscitation Council and an Emeritus Member of the ILCOR BLS Task Force. Maria José C. Carmona receives fees from Cristália Pharma Ind., Medtronic PLC, and União Química Pharma S.A. Editor of the Brazilian Journal of Anaesthesiology. Andrew Lockey is the President of the Resuscitation Council UK, Bernd W. Böttiger is the treasurer of the European Resuscitation Council (ERC); Chairman of the German Resuscitation Council (GRC); Federal Medical Advisor of the German Red Cross (DRK); Member of the Advanced Life Support (ALS) Task Force of the International Liaison Committee on Resuscitation (ILCOR); Member of the Board of the German Interdisciplinary Association for Intensive Care and Emergency Medicine (DIVI), Founder of the ERC Research NET and the German Resuscitation Foundation, Co-Editor of “Resuscitation”; Editor of the Journal “Notfall + Rettungsmedizin”, Co-Editor of the Brazilian Journal of Anaesthesiology. He received fees for lectures from the following companies: Forum für medizinische Fortbildung (FomF), Baxalta Deutschland GmbH, ZOLL Medical Deutschland GmbH, C.R. Bard GmbH, GS Elektromedizinische Geräte G. Stemple GmbH, Novartis Pharma GmbH, Philips GmbH Market DACH, Bioscience Valuation BSV GmbH. Naomi K. Nakagawa is the Brazilian Coordinator of Kids Save Lives Brazil, a Member of the Science and Education Basic Life Support Committee of the European Resuscitation Council, and Co-Editor of Clinics.

## Appendix Supplementary materials

Image, application 1

## References

[1] World Health Organization. Global status report on noncommunicable diseases 2014. https://apps.who.int/iris/handle/10665/148114. Accessed on 4 August 2023.

[2] Chin Y.H., Yaow C.Y.L., Teoh S.E., Foo M.Z.Q., Luo N., Graves N. (2022). Long-term outcomes after out-of-hospital cardiac arrest: a systematic review and meta-analysis. Resuscitation.

[3] Ng T.P., Eng S.W., Ting J.X.R., Bok C., Tay G.Y.H., Kong S.Y.J., GOALS Workgroup (2023). Global prevalence of basic life support training: a systematic review and meta-analysis. Resuscitation.

[4] Hasselqvist-Ax I., Riva G., Herlitz J., Rosenqvist M., Hollenberg J., Nordberg P. (2015). Early cardiopulmonary resuscitation in out-of-hospital cardiac arrest. N Engl J Med.

[5] Huang Q., Hu C., Mao J. (2016). Are chinese students willing to learn and perform bystander cardiopulmonary resuscitation?. J Emerg Med.

[6] Blewer A.L., Ibrahim S.A., Leary M., Dutwin D., McNally B., Anderson M.L. (2017). Cardiopulmonary resuscitation training disparities in the United States. J Am Heart Assoc.

[7] Ko Y.C., Hsieh M.J., Schnaubelt S., Matsuyama T., Cheng A., Greif R. (2023). Disparities in layperson resuscitation education: a scoping review. Am J Emerg Med.

[8] Schroeder D.C., Semeraro F., Greif R., Bray J., Morley P., Parr M. (2023). International liaison committee on resuscitation. KIDS SAVE LIVES: basic life support education for schoolchildren: a narrative review and scientific statement from the International liaison committee on resuscitation. Circulation.

[9] Böttiger B.W., Van Aken H. (2015). Kids Save Lives -training school children in cardiopulmonary resuscitation worldwide is now endorsed by the World Health Organization (WHO). Resuscitation.

[10] Greif R., Bhanji F., Bigham B.L., Bray J., Breckwoldt J., Cheng A. (2020). Education, implementation, and teams: 2020 international consensus on cardiopulmonary resuscitation and emergency cardiovascular care science with treatment recommendations. Education, implementation, and teams collaborators. Circulation.

[11] Greif R., Lockey A., Breckwoldt J., Carmona F., Conaghan P., Kuzovlev A. (2021). European resuscitation council guidelines 2021: education for resuscitation. Resuscitation.

[12] Fleischhackl R., Nuernberger A., Sterz F., Schoenberg C., Urso T., Habart T. (2009). European resuscitation council guidelines 2021: education for resuscitation. School children sufficiently apply life supporting first aid: a prospective investigation. Crit Care.

[13] Abelairas-Gómez C., Rodríguez-Núñez A., Casillas-Cabana M., Romo-Pérez V., Barcala-Furelos R. (2014). Schoolchildren as life savers: at what age do they become strong enough?. Resuscitation.

[14] Breckwoldt J., Beetz D., Schnitzer L., Waskow C., Arntz H.R., Weimann J. (2007). Medical students teaching basic life support to school children as a required element of medical education: a randomised controlled study comparing three different approaches to fifth year medical training in emergency medicine. Resuscitation.

[15] Baldi E., Contri E., Bailoni A., Rendic K., Turcan V., Donchev N. (2019). Final-year medical students' knowledge of cardiac arrest and CPR: we must do more!. Int J Cardiol.

[16] Alkarrash M.S., Shashaa M.N., Kitaz M.N., Rhayim R., Ismail M., Swed S. (2023). Basic life support awareness among medical undergraduate students in Syria, Iraq, and Jordan: a multicenter cross-sectional study. Int J Emerg Med.

[17] Tavares L.F.B., Bezerra I.M.P., Oliveira F.R.S., Souza L.V.A., Raimundo R.D., Souza E.C. (2015). Knowledge of health sciences undergraduate students in objective tests on basic life support. J Hum Growth and Dev.

[18] Lwanga S.K., Lemeshow S. (1991).

[19] Nakagawa N.K., Silva L.M., Oliveira R.C., Oliveira K.M.G., Santos F.R.A., Calderaro M. (2019). KIDS SAVE LIVES BRAZIL: a successful pilot program to implement CPR at primary and high schools in Brazil, resulting in state law for a training CPR week. Resuscitation.

[20] Lu C., Jin Y., Meng F., Wang Y., Shi X., Ma W. (2016). An exploration of attitudes toward bystander cardiopulmonary resuscitation in University students in Tianjin, China: a survey. Int Emerg Nurs.

[21] Contri E., Bonomo M.C., Costantini G., Manera M., Bormetti M., Tonani M. (2017). Are final year medical students ready to save lives in Italy? Not yet. Emerg Med J.

[22] Brenner P.S., DeLamater J. (2016). Lies, damned lies, and survey self-reports? Identity as a cause of measurement bias. Soc Psychol Q.

