# Supplementary material for: Willingness and skills among students from non-health academic fields in providing efficient basic life support
Source: Clinics (Sao Paulo). 2024 Nov 8;79:100518. doi: 10.1016/j.clinsp.2024.100518 (PMC11583723; doi:10.1016/j.clinsp.2024.100518)
Supplement: Supplementary file 1 [file mmc1.docx]

**CLINICS-D-24-00359_ Supplementary Material**

**Supplementary Material**

|  | **3 points** | **2 points** | **1 point** | **0 point** |
| --- | --- | --- | --- | --- |
| **KNOWLEDGE** |  |  |  |  |
| 1. Do you know what the emergency number is in Brazil? | ‒ | 192 or 911 | ‒ | Wrong answers |
| 2. Do you know what an automatic external defibrillator (AED) is? | ‒ | It is a device to apply shock | ‒ | Wrong answers |
| 3. Do you know what is a cardiac arrest? | ‒ | ‒ | Yes | Little or no |
| 4. Do you know what is acute myocardial infarction? | ‒ | ‒ | Yes | Little or no |
| 5. Do you know what is cardiopulmonary resuscitation? | ‒ | ‒ | Yes | Little or no |
| **SKILLS** |  |  |  |  |
| 1. Have you already learned how to act in a sudden cardiac arrest? | ‒ | Yes, I learned it at school or BLS course | ‒ | No or in films, talks etc. |
| 2. If a person falls unconscious in front of you, what would you do first? | ‒ | Verify scene safety | ‒ | I don’t know or other answers |
| 3. What are the signs of life to check in a victim that suddenly fall? | ‒ | Responsiveness and breathing | ‒ | I don’t know or other answers |
| 4. When should you call an emergency medical service? | ‒ | When victim doesn't move, speak or open eyes and/or breathe | ‒ | I don’t know or other answers |
| 5. Are you able to perform effective chest compressions on the victim following telephone instructions from the medical emergency service? | ‒ | Yes | A little | No |
| 6. Do you know what position the victim must be to receive effective chest compressions? | ‒ | ‒ | Horizontal dorsal | No or other answers |
| 7. Do you know how many chest compressions should be performed per minute? | 100‒120 compressions | ‒ | ‒ | No or other answers |
| 8. Do you know how to use an automatic external defibrillator? | ‒ | Yes | A little | No |
| **ATTITUDE** |  |  |  |  |
| 1. Would you help a victim of cardiac arrest? | ‒ | ‒ | Maybe | No or I don’t know |
| 2. Do you find useful to know how to resuscitate a victim? | ‒ | ‒ | Yes | No or I don’t know |
| 3. Have you ever witnessed a situation of sudden cardiac arrest? | ‒ | ‒ | 1, 2 or several | No or I don’t know |
| 4. Would you help a victim of acute myocardial infarction? | ‒ | ‒ | Yes | No or I don’t know |
| 5. Do you find useful to know how to act in acute myocardial infarction situation? | ‒ | ‒ | Yes | No or I don’t know |
| 6. Have you ever witnessed a situation of acute myocardial infarction? | ‒ | ‒ | 1, 2 or several | No or I don’t know |
| 7. Do you think that your school should give practical training on how to recognize and act in a sudden cardiac arrest situation? | ‒ | Yes, annually | Yes, a little | No or I don’t know |
| 8. Do you think that your school should give practical training on how to recognize and to act in acute myocardial infarction situations? | ‒ | Yes, annually | Yes, a little | No or I don’t know |
| 9. Do you think that your school should give mandatory practical training on how to recognize and to act in a sudden cardiac arrest situation? | ‒ | Yes, annually | Yes, a little | No or I don’t know |
| 10. Do you think that your school should give mandatory practical training on how to recognize and to act in acute myocardial infarction? | ‒ | Yes, annually | Yes, a little | No or I don’t know |
| 11. Would you like to learn in a 4-hour practical course for laypeople how to recognize and to act in sudden cardiac arrest situation? | ‒ | ‒ | Yes | No or I don’t know |
| 12. Would you like to learn in a 4-hour practical course for laypeople how to recognize and to act in acute myocardial infarction situation? | ‒ | ‒ | Yes | No or I don’t know |
